# ICTV Virus Taxonomy Profile: *Plectroviridae*

**DOI:** 10.1099/jgv.0.001597

**Published:** 2021-05-07

**Authors:** Petar Knezevic, Evelien M. Adriaenssens, ICTV Report Consortium

**Affiliations:** ^1^​ University of Novi Sad, Faculty of Sciences, Department of Biology and Ecology, Trg Dositeja Obradovica 3, 21000 Novi Sad, Vojvodina, Serbia; ^2^​ Quadram Institute Bioscience, Norwich Research Park, Norwich NR4 7UQ, UK

**Keywords:** *Plectroviridae*, ICTV, taxonomy

## Abstract

Members of the family *Plectroviridae* produce particles that are non-enveloped rigid rods (70–280×10–16 nm). The supercoiled, circular, single-stranded DNA genome of about 4.5–8.3 kb, encodes 4–13 proteins. Viruses of this family infect cell wall-less bacteria, adsorbing to the bacterial surface, replicating their DNA by a rolling-circle mechanism or transposition, and releasing progeny from cells by extrusion, without killing the host. This is a summary of the International Committee on Taxonomy of Viruses (ICTV) Report on the family *Plectroviridae* which is available at ictv.global/report/plectroviridae.

## Virion

**Fig. 1.**
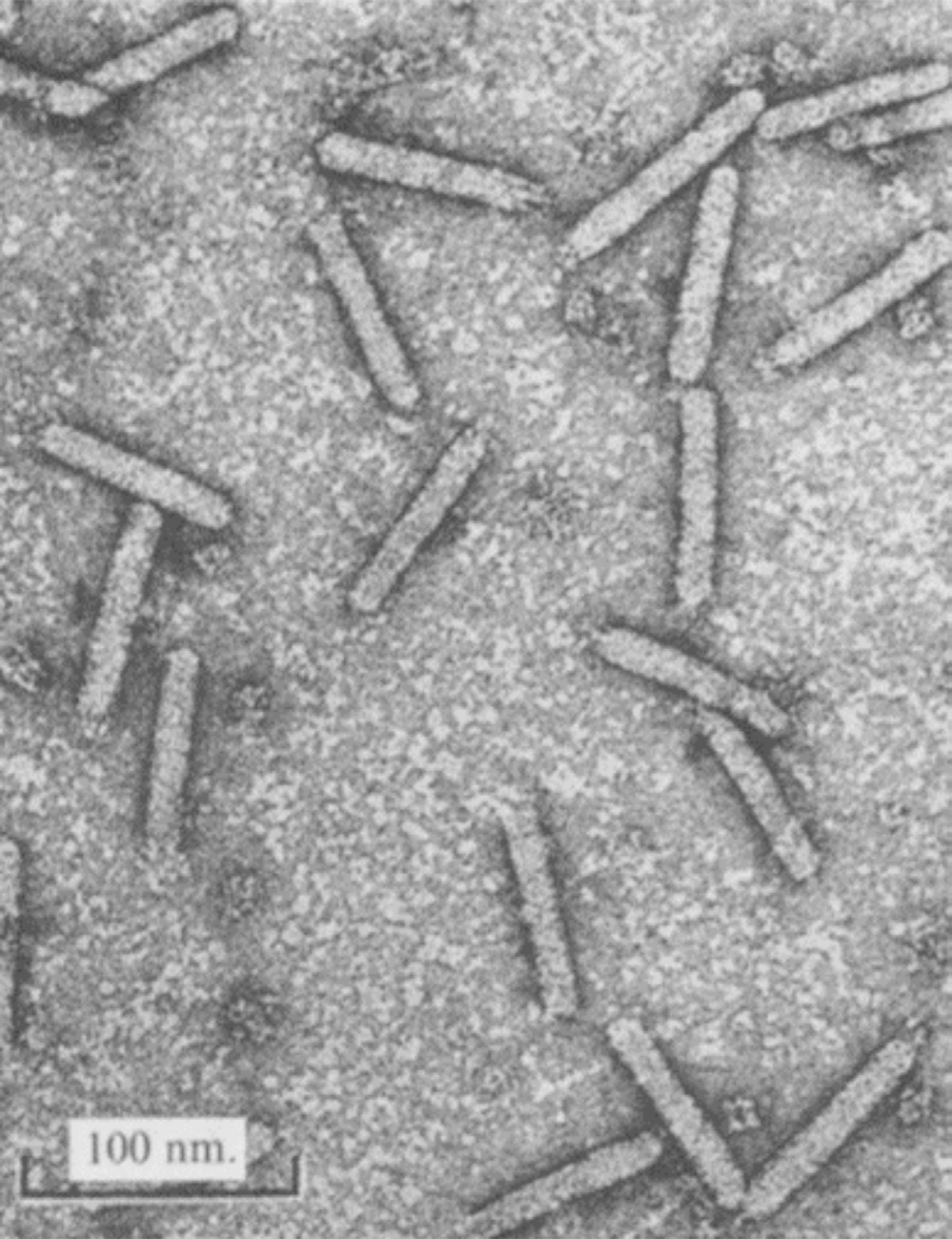
Acholeplasma phage MV-L1, negatively-stained with 2 % uranyl acetate (from [3] with permission).

Virions of members of the family *Plectroviridae* are rigid, asymmetric, non-enveloped, nearly straight rods with one end rounded, and the other more variable (Table 1, Fig. 1). Acholeplasma phages (genus *Plectrovirus*) are 70–90 nm long and 14–16 nm in diameter, whereas several phages that infect *Spiroplasma* (genera *Vespertiliovirus* and *Suturavirus*) are 230–280 nm long and 10–16 nm wide [1–3], being shorter and wider than members of the family *Inoviridae* and *Paulinoviridae*, the other families in the order *Tubulavirales*.

**Table 1.** Characteristics of members of the family *Plectroviridae*

Example:	Acholeplasma phage MV-L1 (X58839), species *Acholeplasma virus L51,* genus *Plectrovirus*
Virion	Non-enveloped rigid rods; 10–16 nm in diameter, 70–280 nm in length
Genome	4.5–8.3 kb, supercoiled, circular, positive-sense single-stranded DNA; 4–13 encoded proteins
Replication	Rolling-circle replication or transposition
Translation	From mRNAs
Host range	Cell wall-less bacteria
Taxonomy	Realm *Monodnaviria*; kingdom: *Loebvirae*; phylum *Hofneiviricota*; class *Faserviricetes*, order *Tubulavirales*: the family *Plectroviridae* includes several genera and species

## Genome

**Fig. 2.**
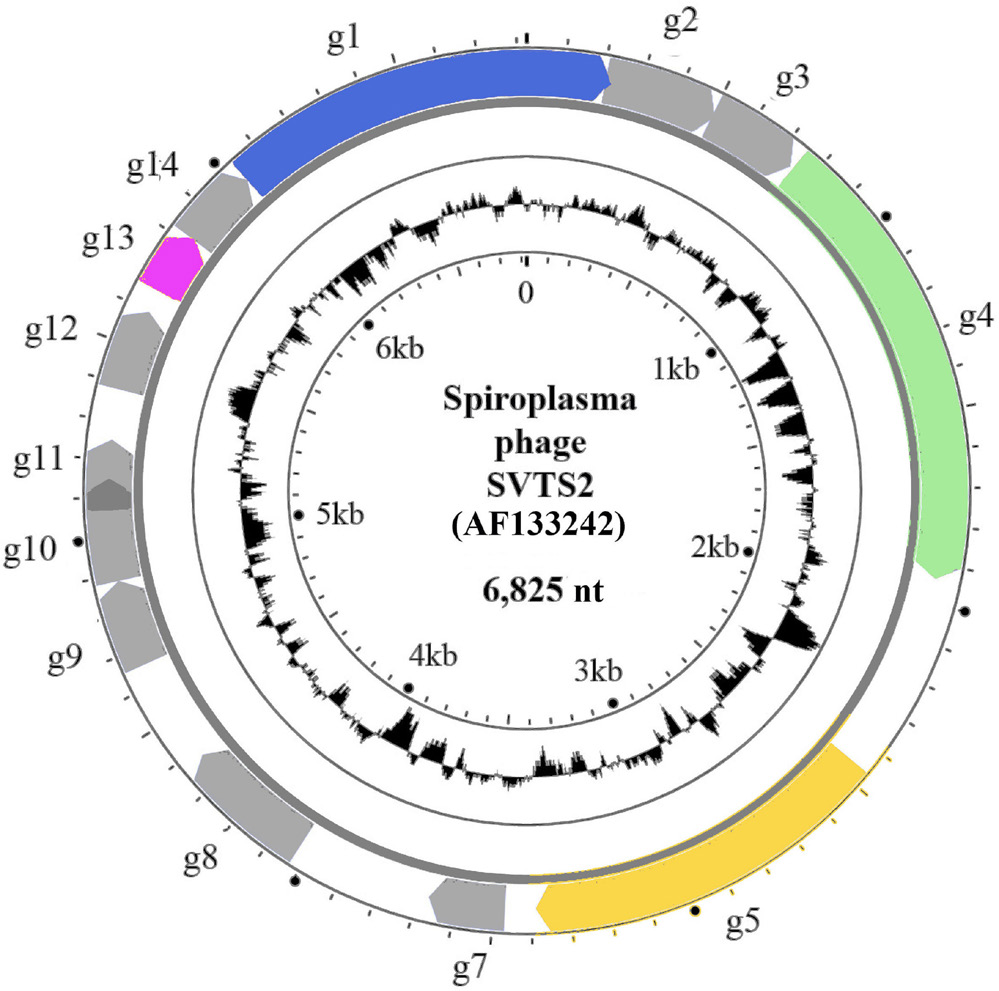
Genome organisation of Spiroplasma phage SVTS2. The outer circle shows the circular genome (AF133242) with genes g1–14 indicated by coloured arrows (g10 and g11 overlap, there is no g6). Genes with known products are coloured: g1 - adhesion protein (CoaA), g4 - maturation (morphogenesis, assembly) protein, g5 - replication protein and g13 - major coat protein (CoaB). The inner circle represents GC content.

Nucleic acid present in virions consists of a supercoiled, circular, positive-sense single-stranded DNA molecule (Fig. 2). Genomes range from 4.5 kb in Acholeplasma phage MV-L1 to 8.3 kb in Spiroplasma phage 1-R8A2B, with the number of encoded proteins ranging from 4 to 13. Most predicted proteins have unknown functions, and so it is not known if there is a modular genome organization similar to that described for members of the family *Inoviridae*. The G+C content varies from 22.2 % for Spiroplasma phage SkV1CR23x to 33.3 % for Acholeplasma phage MV-L51.

## Replication

Virions adhere to the surface of cell wall-less bacteria, the receptor for Acholeplasma phage MV-L1 being protein-lipoglycane molecules [4]. Replication of the positive-sense single-stranded DNA genome is by a rolling-circle mechanism for members of the genus *Plectrovirus* and, by inference, for members of the genus *Suturavirus*. Phages in the genus *Vespertilliovirus* possess a transposase gene instead of an equivalent to p2 of Escherichia phage M13 (*Inoviridae*), and use a transposition mechanism, with the encapsidated genome representing a circular transposition intermediate [5]. A specific trait of phages belonging to the genera *Suturavirus* and *Vespertiliovirus* is that UGA in mRNAs is not a stop codon but, as in the host bacterium, encodes tryptophan [6].

Virions assemble and are released at the host membrane by extrusion while the host cells continue to divide.

## Taxonomy

Current taxonomy: www.ictv.global/taxonomy. The family *Plectroviridae* belongs to the order *Tubulavirales*. Members of the same genus share considerable similarity of DNA sequences, and >50 % similarity (identiity×query coverage) of the major coat protein (CoaB) and maturation protein amino acid sequences. Phages of the same species share >95 % DNA sequence identity over the complete genome and significant amino-acid sequence similarity of the adhesion protein (CoaA).

## Resources

Full ICTV Report on the family *Plectroviridae*: www.ictv.global/report/plectroviridae.
